# Effect of Olive Pomace Extract Application and Packaging Material on the Preservation of Fresh-Cut Royal Gala Apples

**DOI:** 10.3390/foods12091926

**Published:** 2023-05-08

**Authors:** Joana Madureira, Bruno Melgar, Vítor D. Alves, Margarida Moldão-Martins, Fernanda M. A. Margaça, Celestino Santos-Buelga, Lillian Barros, Sandra Cabo Verde

**Affiliations:** 1 Centro de Ciências e Tecnologias Nucleares (C2TN), Instituto Superior Técnico, Universidade de Lisboa, 2695-066 Loures, Portugal; joanamadureira@ctn.tecnico.ulisboa.pt (J.M.); fmargaca@ctn.tecnico.ulisboa.pt (F.M.A.M.); sandracv@ctn.tecnico.ulisboa.pt (S.C.V.); 2 Centro de Investigação de Montanha (CIMO), Instituto Politécnico de Bragança, Campus de Santa Apolónia, 5300-253 Bragança, Portugal; bruno.melgarc@ipb.pt; 3 Laboratório Associado para a Sustentabilidade e Tecnologia em Regiões de Montanha (SusTEC), Instituto Politécnico de Bragança, Campus de Santa Apolónia, 5300-253 Bragança, Portugal; 4 Grupo de Investigación en Polifenoles (GIP-USAL), Facultad de Farmacia, Universidad de Salamanca, Campus Miguel de Unamuno, 37007 Salamanca, Spain; csb@usal.es; 5 LEAF—Linking, Landscape, Environment, Agriculture and Food—Research Center, Instituto Superior de Agronomia (ISA), Universidade de Lisboa, Tapada da Ajuda, 1349-017 Lisbon, Portugal; mmoldao@isa.ulisboa.pt; 6 Departamento de Engenharia e Ciências Nucleares, Instituto Superior Técnico, Universidade de Lisboa, 2695-066 Bobadela LRS, Portugal; 7 Unidad de Excelencia Producción, Agrícola y Medioambiente (Agrienvironment), Parque Científico, Universidad de Salamanca, 37185 Salamanca, Spain

**Keywords:** fresh-cut apples, storage, shelf life, natural extracts, packaging films, microbial quality

## Abstract

The efficiency of natural olive pomace extracts for enhancing the quality of fresh-cut apples was compared with commercial ascorbic acid and two different packaging films (biodegradable polylactic acid (PLA) and oriented polypropylene (OPP)) were tested. The composition of atmosphere inside the packages, the physicochemical parameters (firmness, weight loss and color), the microbial load, total phenolic content and antioxidant activity of fresh-cut apples were evaluated throughout 12 days of storage at 4 °C. After 12 days of refrigerated storage, a significant decrease in O_2_ was promoted in PLA films, and the weight loss of the whole packaging was higher in PLA films (5.4%) than in OPP films (0.2%). Natural olive pomace extracts reduced the load of mesophilic bacteria (3.4 ± 0.1 log CFU/g and 2.4 ± 0.1 log CFU/g for OPP and PLA films, respectively) and filamentous fungi (3.3 ± 0.1 log CFU/g and 2.44 ± 0.05 log CFU/g for OPP and PLA films, respectively) growth in fresh-cut apples after five days of storage at 4 °C, and no detection of coliforms was verified throughout the 12 days of storage. In general, the olive pomace extract preserved or improved the total phenolic index and antioxidant potential of the fruit, without significant changes in their firmness. Moreover, this extract seemed to be more effective when combined with the biodegradable PLA film packaging. This work can contribute to the availability of effective natural food additives, the sustainability of the olive oil industries and the reduction of environmental impact. It can also be useful in meeting the food industries requirements to develop new functional food products.

## 1. Introduction

In recent years, changes in family lifestyles and growing health concerns among consumers have led to a growing demand for minimally processed foods, increasing market growth worldwide. Consumers are looking for fresh-like processed products, such as fruits, with high quality attributes (appearance, texture and flavor, among others) to satisfy their daily needs of antioxidants, minerals and dietary fibers [1].

Fruits, such as apples, are an excellent source of vitamins, minerals, fibers and antioxidants that are essential to reduce the risk of developing heart disease, cancer, inflammation and diabetes. In Portugal, apples are one of the most important fruit markets, with a consumption per capita that reached 30.5 kg in 2021 [2]. One of the main challenges of the fruit industries, including apple processing, is to maintain the post-harvest quality during storage, distribution and sale to the consumer in order to ensure that the fruit remains healthy and safe [3]. Minimal fruit processing can result in quality deterioration due to increased respiration rate, ethylene production and cut-surface browning, which implies water loss, softening and microbial contamination. As expected, minimally processed apples are more perishable than unprocessed whole apples, especially due to the break or elimination of natural protection systems and the susceptibility for undesirable enzymatic browning promoted by the oxidation of phenolic compounds by polyphenol oxidase, peroxidase or tyrosinase [4]. Moreover, yeast fermentation and mold spoilage can also occur on the surface of the slice [5]. Sulfites have been widely used as antibrowning agents, however, many adverse reactions to human health have been associated to their use, and they are forbidden by the Food and Drug Administration [6]. Thus, ascorbic acid, citric acid and some sulfur-containing amino acids have been introduced alone or in combination with firming or antimicrobial agents [7]. Recently, in order to replace synthetic antioxidants, natural additives from have been incorporated in food, such as meat, dairy and bakery products [8,9,10,11]. Olive wastes are considered valuable sources of natural phenolic compounds with health benefits, such as hydroxytyrosol, tyrosol, oleuropein and verbascoside [12,13], have been used as food ingredients to enrich bread, biscuits, pasta and meat [14,15,16,17,18,19]. Madureira et al. [20] and Difonzo et al. [21] reviewed the main applications of the phenolic compounds from olive pomace with high added value in food products. Lin et al. [17] used incorporated olive pomace extracts into biscuits and observed higher fiber abundance, nutritional quality and acceptability and lower calories and glycemic index. Additionally, Galanakis et al. [14] observed higher antimicrobial activity and extended shelf life of bread and rusks from 10 to 15 days when fortified with olive polyphenols (200 mg of polyphenols/kg), while the addition of olive leaf extracts to poultry meat reduced microbial growth while maintaining both chemical quality and sensory attributes, and extending the shelf life of the meat when refrigerated for 15 days [15].

The packaging is also very important to slow the respiration rate and prevent the growth of aerobic spoilage microorganisms in food products [5]. The most used food packaging materials are petroleum-based polymers such as polypropylene (PP) due to their low-cost and good barrier performances. These plastics are non-biodegradable and non-renewable, causing human and environmental risks [22]. Therefore, in recent years, special attention has been focused on biodegradable food packaging in search of alternative materials to replace these plastics. Biodegradable products are mainly produced from biopolymers, such as cellulose, chitosan, starch, polylactic acid (PLA), collagen and casein. To be used in the food industry, these materials must be non-toxic, renewable and possessing specific properties [23]. PLA is a biopolymer produced by bacterial fermentation of renewable resources such as corn or sugar beets [23], with the advantage of being compostable and biocompatible and having high mechanical strength [22].

Ionizing radiation is a clean and environmentally friendly technology that does not require the addition of chemicals. It is evidenced to be capable of enhancing the phenolic compounds extraction and antioxidant activity on industrial wastewater [24], fresh fruits such as cherry tomatoes [25], raspberries [26], and strawberries [27] and dried medicinal plants [28]. More recently, the authors demonstrated the suitability of gamma radiation at 5 kGy to improve the extractability of phenolic compounds from olive pomace by two-fold compared to the non-irradiated samples [12]. The optimized conditions to obtain extracts with higher bioactivities were also achieved using heat-assisted extraction [13]. Among other properties, these natural extracts presented higher antioxidant and antimicrobial potentials, which could indicate the possibility of using them as alternatives to synthetic food preservatives.

The aim of this work was to evaluate the use of irradiated olive pomace extracts as natural ingredients to extend shelf life of fresh-cut apples by maintaining quality attributes and increasing bioactive properties. Two different packaging films (biodegradable polylactic acid (PLA), and oriented polypropylene (OPP)) were used to compare both performances. The comparison with ascorbic acid, a commercial antioxidant used by food industries, was assessed, and physicochemical parameters (firmness, weight loss and color), atmospheric composition inside the packages and the microbial load throughout 12 days of storage at 4 °C were evaluated. Moreover, the phenolic content and antioxidant activity of the fruits were assessed throughout the 12 days of storage. As far as the authors know, this is the first study to use an extract of this agro-industrial residue as additive for fresh-cut fruits to enhance their quality. The obtained results could also represent a strategy to promote the sustainability of the olive oil industry and contribute to the promotion of a circular economy.

## 2. Materials and Methods

### 2.1. Chemicals

Methanol and ethanol were acquired from Panreac AppliChem (Darmstadt, Germany) and Honeywell (Charlotte, NC, USA), respectively. Gallic acid, iron chloride, Folin-Ciocalteau reagent and 2,2-diphenyl-1-picrylhydrazyl (DPPH) were purchased from Sigma-Aldrich (St. Louis, MO, USA), and ascorbic acid and ferrous sulphate were obtained from Merck (Darmstadt, Germany). Acetic acid, hydrochloric acid and sodium acetate were provided by Honeywell-Riedel-de Haën (Charlotte, NC, USA). Sodium carbonate was acquired from JMGS (Odivelas, Portugal), and 2,4,6-Tris(2-pyridyl)-s-triazine (TPTZ) was obtained from Honeywell-Fluka (Charlotte, NC, USA). Tryptic Soy Agar (TSA), Malt Extract Agar (MEA) and Violet Red Bile Agar (VRBA) were obtained from Oxoid—Thermo Scientific (Waltham, MA, USA). Water was treated in a Milli-Q water purification system (Merck Millipore, Burlington, MA, USA).

### 2.2. Olive Pomace Samples and Irradiation Experiments

The samples used in this work were olive pomaces collected in November 2020 from UCASUL (União de Cooperativas Agrícolas do Sul, located in the Alentejo region, Portugal) and further subjected to gamma radiation treatment in a Co-60 semi-industrial unit (with an activity of 126 kCi in March 2021) located at Technological Unit of Radiosterilization (UTR-IST), University of Lisbon (Portugal). Sealed bags (20 cm × 10 cm) containing 100 g of extracted olive pomace were irradiated at room temperature at 5.2 ± 0.2 kGy using a dose rate of 10.4 kGy/h. The absorbed doses were measured using Amber Perspex routine dosimeters [29] (dose uniformity DUR = 1.07). The irradiation experiments were performed in triplicate.

**Figure 1:**
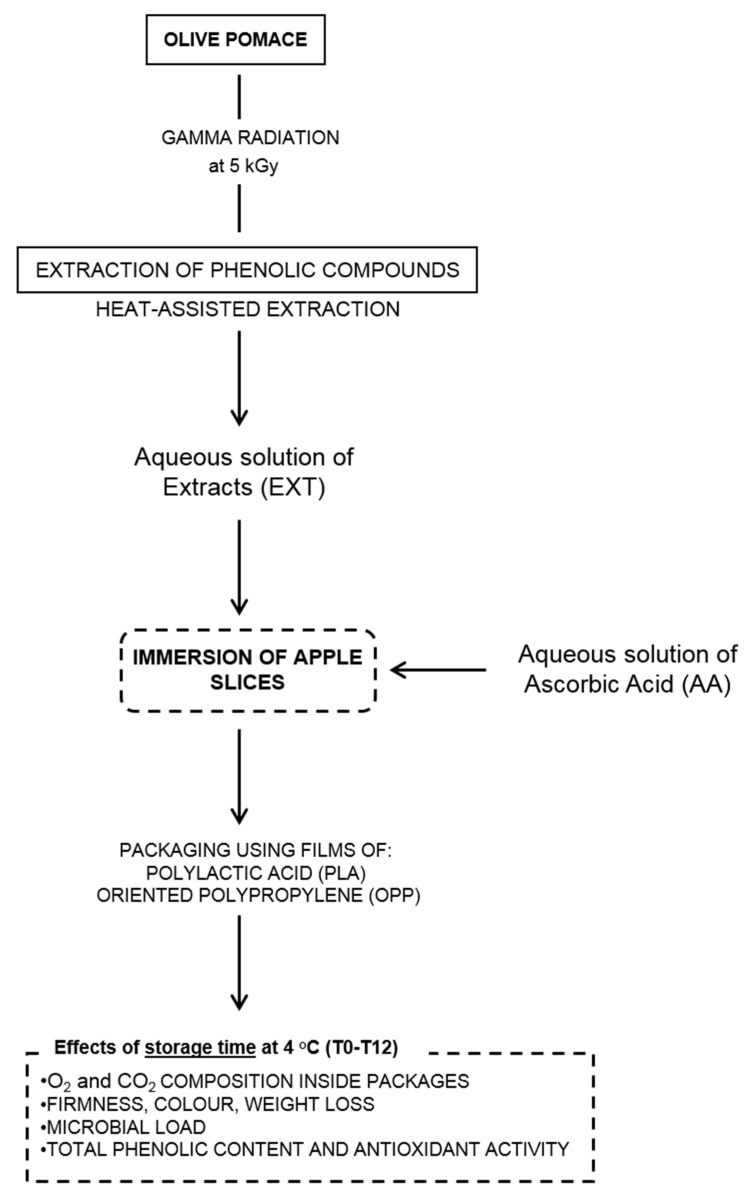
Schematic diagram of the experimental procedure of this work.

A schematic diagram of the experimental steps performed in this research is shown in Figure 1.

### 2.3. Olive Pomace Natural Ingredients: Phenolic Extract Preparation

After gamma radiation, the phenolic compounds from olive pomace were extracted using the optimal conditions of heat-assisted extraction explored by Madureira et al. [30]. Briefly, 7 g of olive pomace were extracted in 240 mL of 76% ethanol for 120 min at 85 °C. These extraction conditions were selected due to the improved bioactive properties obtained for these extracts that were evaluated in a previous work of the authors [13]. The extractions were performed in triplicate.

### 2.4. Polylactic Acid (PLA) and Oriented Polypropylene (OPP) Films Packages

The film materials used to produce the packages for this study were polylactic acid (PLA) and oriented polypropylene (OPP).

PLA films in bag form were manufactured by the company Vegware (Edinburgh, UK). These biodegradable films were produced from corn starch and included a transparent side (thickness = 0.029 mm) and an opaque white side (thickness = 0.039 mm). The films had a permeability to O_2_ of 2.97 × 10^−16^ m^2^/s, a permeability to CO_2_ of 1.22 × 10^−15^ m^2^/s and a permeability to water vapor of 1.10 × 10^−12^ mol·m/m^2^·s·Pa.

Conventional commercial film made of OPP was supplied by Campotec S.A (Torres Verdras, Portugal) and manufactured by Pigmea S.L. (Jaén, Spain). This film has a thickness of 0.030 mm, a permeability to O_2_ of 2.68 × 10^−12^ m^2^/s, and a permeability to water vapor of 8.75 × 10^−14^ mol·m/m^2^·s·Pa.

Both types of film bags (10 × 10 cm) were manually constructed and further used as described below.

### 2.5. Preparation of Fresh-Cut Apples

Apples (Royal Gala variety) of uniform shape and size were purchased from a local supermarket in Lisbon, Portugal, and immediately stored at 4 ± 1 °C until analysis. The fruits were washed/disinfected with sodium hypochlorite solution (60 mg free chlorine/L) for 1 min and air-dried. Then, apples were cut with a disinfected kitchen knife into equal slices (8 slices per apple), and three groups of samples were prepared by immersion for one minute. The control samples were immersed in water, one experimental group was immersed in olive pomace extract solution (0.315%, *w*/*v*) and a second group was immersed in ascorbic acid solution (0.315%, *w*/*v*). A concentration of 0.315% *w*/*v* was applied to the samples based on preliminary experiments demonstrating that this concentration presented the highest antioxidant activity without significantly changing the color of apple slices upon application. After immersion in their respective solutions, the samples were air-dried and sealed in PLA or OPP film bags, then stored in controlled chambers at 4 °C and 85% relative humidity.

The fruits were analyzed over time (after 0, 2, 5, 7, 9 and 12 days of storage at 4 °C), in terms of surface color, weight loss, firmness, microbial load, phenolic content and antioxidant activity. The composition of the atmosphere inside the fruit packages was also measured over time. Three independent packages were analyzed for each group on each day. For physicochemical parameters (firmness, color and weight loss) and package gas composition, the control samples were fruits immersed in ascorbic acid (PLA-AA and OPP-AA). For antioxidant activity and microbial load evaluation, the control samples were apple slices with no treatment (PLA-NT and OPP-NT).

### 2.6. Analytical Methods

#### 2.6.1. Soluble Solids Content, Titratable Acidity and Respiration Rate

In the initial stage of the work, a physicochemical characterization of the apple samples was performed. Total soluble solids (TSS) and titratable acidity (TA) were determined for apples using the pulp of four fruits. TSS measurements (average of six measurements) were performed with a hand-held refractometer ATAGO (Atago Co, Ltd., Tokyo, Japan), and the results were expressed as °Bx. TA was determined by titration and expressed as % of malic acid (average of three measurements). Respiration rate (RT; mmol CO_2_/kg·h) was measured using a gas analyzer (Checkmate 9900, PBI Dansensor, Ringsted, Denmark), as described by Vieira, Moldão-Martins and Alves [31].

#### 2.6.2. Composition of the Atmosphere Inside the Packages

The concentration of carbon dioxide (CO_2_) and oxygen (O_2_) inside the packages was measured using a gas analyzer (Checkmate 9900, PBI Dansensor, Ringsted, Denmark) [32]. An adhesive silicon septum was glued to the sampling point of the packages to prevent gas leakage during analysis. The needle of the gas analyzer was inserted through the septum, and results were expressed in percentages of O_2_ and CO_2_. Three packages per group were analyzed on each test day.

#### 2.6.3. Weight Loss, Firmness and Surface Color

The physicochemical parameters were measured according to Vieira et al. [32]. The weight loss (% from the original weight) from each sealed tray was measured using an electronic balance (TC-403, Denver Instrument Company, Vernon Hills, IL, USA). The results were expressed as an average of three replicates per group for each test day.

A Texturometer (TA-XT2, Stable Micro System, Surrey, UK) with a 5 kg load cell was equipped with a flat probe (37 mm diameter) and used to evaluate the firmness of the apple slices [32]. A total of 9 slices from each package and each group (per test day) were assayed. Each fruit was positioned under the probe and compressed to 80% deformation at a speed of 1 mm/s.

A Konica Minolta CTR-300 colorimeter (Minolta, Williams Drive Ramsey, NJ, USA) was used to measure L* (lightness), a* (red–green) and b* (yellow–blue) color parameters of apple slices. The colorimeter was calibrated using a standard white plate provided by the manufacturer. The color of the samples was measured on both sides of the apple slices from each package and each group on each test day (a total of 18 measurements per package and group). Furthermore, the color of the apple slices was expressed as the hue angle (h°), total color differences (∆E) and chroma (C) related to saturation [31], which were calculated from the parameters represented by the following Equations (1)–(5):

(1) $$C^{*}={\left(a^{*2}+b^{*2}\right)}^{1/2}$$

(2) $$h{}^{\circ}=arctan\left(\;\frac{b^{*}}{a^{*}}\right)\times \frac{180}{\Pi },\;\mathrm{if}\;a^{*}\;>\;0\;\mathrm{and}\;b^{*}\;>\;0$$

(3) $$h{}^{\circ}=arctan\left(\;\frac{b^{*}}{a^{*}}\right)\times \frac{180}{\Pi }+180,\;\mathrm{if}\;a^{*}\;<\;0$$

(4) $$h{}^{\circ}=arctan\left(\;\frac{b^{*}}{a^{*}}\right)\times \frac{180}{\Pi }+360,\;\mathrm{if}\;a^{*}\;>\;0\;\mathrm{and}\;b^{*}\;<\;0$$

(5) $$\Delta E={\left[{\left(\Delta L\right)}^{2}+{\left(\Delta a^{*}\right)}^{2}+{\left(\Delta b^{*}\right)}^{2}\right]}^{1/2}\;$$

#### 2.6.4. Microbial Load

Apple slices from each package (~150 g) were placed in sterile stomacher bags containing 100 mL of buffered peptone water. Samples (n = 3, per each package, group and test day) were homogenized using a stomacher (Stomacher 3500; Seaward, UK) for 15 min. Serial decimal dilutions were prepared for inoculation in triplicate on Tryptic Soy Agar plates (TSA) for mesophilic microbial counts, Malt Extract Agar (MEA) plates for filamentous fungi counts and Violet Red Blue Agar (VRBA) plates for coliforms counts. TSA plates were incubated at 30 °C, MEA plates at 25 °C and VRBA plates at 37 °C. The colony numbers were counted for 7 days. The results were expressed as the log of colony-forming units per gram of fresh fruit (log CFU/g).

#### 2.6.5. Total Phenolic Content and Antioxidant Activity

For the quantification of the total phenolic content and the antioxidant activity, extracts were obtained from freeze-dried apple slices using the same methodology described by Madureira et al. [12], with an ethanol:water mixture (80:20 *v*/*v*) used as a solvent. The dry extracts were obtained after lyophilisation and used for all the analyses.

Total Phenolic Content (TP) was determined using the Folin-Ciocalteau method [33] using extract solutions with a concentration of 20 mg/mL. Absorbance was measured at 765 nm using a spectrophotometer (Shimadzu UV 1800, Kyoto, Japan), and the results were expressed as mg of gallic acid equivalents (GAE) per gram of extract. The assay was carried out in triplicate for each package, group and test day.

Antioxidant activity was evaluated using two assays: DPPH radical scavenging activity described by Brand-Williams, Cuvelier and Berset [34] with some modifications, and Ferric Reducing Antioxidant Power (FRAP) described by Benzie and Strain [35]. For the DPPH method, the extracts were dissolved in distilled water at a concentration of 40 mg/mL and then successive dilutions were prepared (40–1.25 mg/mL). The reduction of the DPPH radical was determined by measuring the absorption at 515 nm using an EZ Read 1200 Microplate Reader (Biochrom, Cambridge, UK). The radical scavenging activity (RSA) was calculated as a percentage of DPPH discoloration using the following equation: %RSA = [(A_DPPH_ − A_S_)/A_DPPH_] × 100, where A_S_ is the absorbance of the solution when the sample extract had been added and A_DPPH_ is the absorbance of the DPPH solution. Ascorbic acid was used as standard. The results were expressed in terms of IC_50_ values (mg/mL), indicating the extract concentrations that provided 50% of antioxidant activity [36]. For FRAP assay, the extracts were dissolved in distilled water at a concentration of 5 mg/mL. The reduction of the ferric ion (Fe^3+^)–ligand complex to the intensely blue-colored ferrous (Fe^2+^) was measured at 593 nm using a spectrophotometer (Shimadzu UV 1800, Kyoto, Japan). The results were expressed as mmol of ferrous sulfate equivalent (FSE) per gram of extract. Both assays were performed in triplicate for each package, group and test day.

### 2.7. Statistical Analysis

Data were expressed as means ± standard error. For statistical analysis, confidence intervals for mean values were estimated considering a significance level of *p* < 0.05 and the number of replicates for each assay. The differences among treatments were analyzed using a one-way analysis of variance (ANOVA) followed by Tukey’s HSD test with α = 0.05.

### 2.8. Principal Components and Overlayed K-Means Clustering

The data collected from the previous analysis (included in the repository uploaded as Supplementary Materials to this work) were subjected first to mean transformation (replicate measurements) and then to data imputation using the following approaches: (1) considering the means of different observations when the data were scarce; (2) interpolations of data were performed using linear regressions; (3) one extrapolation was made using a polynomial regression employing data from similar conditions to avoid over- or under-fitting of the extrapolated value. Afterwards, the imputed dataset was used for the principal component analysis (PCA) computation considering 3 principal components represented on 2 dimensions using a conversion of the 3rd PC to factorial data. Additionally, after a first approach considering all the responses, lower variability responses (L*, a*, b*, C, firmness, DPPH and FRAP) were removed and processed again through PCA. Then, K-means clustering was overlaid on the PCA data in order to compute the optimal number of clusters relying in the average silhouette width method. The software employed was R studio version 2022.12.0 + 353. The packages and scripts are also provided in the Supplementary Materials.

## 3. Results and Discussion

### 3.1. Physicochemical Characterization of the Samples

The titratable acidity (TA) and the total soluble solids (TSS) of the apple samples were 0.23% malic acid and 12.2 °Bx, respectively, which are in accordance with the results reported in the literature for different cultivars of apples and different processing fractions [37,38,39,40,41,42,43,44]. The respiration rate was assessed by evaluating the CO_2_ production, which can provide essential information about the fruit’s metabolic activity. In this work, the apple slices in cold storage (4 °C) showed a moderate respiration rate (11 mmol CO_2_/kg·h), which significantly decreased with increasing storage time.

### 3.2. Composition of the Atmosphere Inside the Package

**Figure 2:**
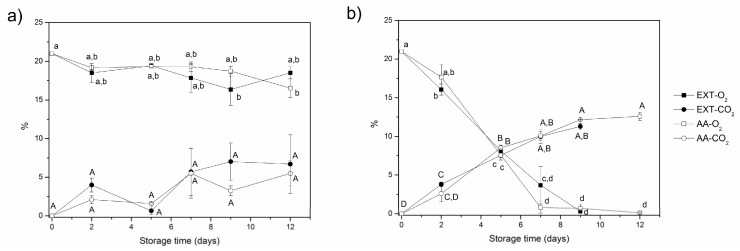
Variation of headspace gas composition (% O_2_ and % CO_2_) inside the packages of apple slices stored for 12 days at 4 °C in: (**a**) OPP films and (**b**) PLA films (OPP—oriented polypropylene; PLA—polylactic acid; EXT—extract of olive pomace; AA—ascorbic acid). Vertical bars indicate 95% confidence intervals of three replicates. Means with equal letters (uppercase letters for CO_2_ and lowercase letters for O_2_) are not statistically different by Tukey’s test with a 5% significance level.

The O_2_ and CO_2_ contents inside the packages were recorded throughout the storage time (Figure 2).

Considering the internal volume (81 mL) that was not occupied by the apples to be constant, the variation in the atmosphere inside the packages was created by the respiration rate of the fruits, the metabolic activity of microbial development and the permeability of the films to O_2_ and CO_2_, which can be affected by the temperature. As expected, a decrease in the headspace O_2_ and an increase in the headspace CO_2_ during the storage time were observed (Figure 2). In general, no significant differences (*p* > 0.05) were observed when comparing the samples treated with olive extracts and ascorbic acid for each film packaging: OPP (Figure 2a) or PLA (Figure 2b). Nevertheless, the atmosphere composition during storage at 4 °C varied depending on the film used. The content of O_2_ inside the OPP packages did not significantly change (*p* > 0.05) during the storage time, whereas for the PLA film, a marked decrease was noticeable with storage, and it was significantly different (*p* < 0.05) from the OPP film after five days of storage. The same trend was observed for the CO_2_ content. No significant differences (*p* > 0.05) were detected in CO_2_ content throughout the storage time when using OPP films (Figure 2a), whereas the PLA films promoted a significant increase in CO_2_ content (Figure 2b). These results revealed that PLA films are poorly permeable to gases, confirming their lower permeability to O_2_ (2.97 × 10^−16^ m^2^/s) and CO_2_ (1.22 × 10^−15^ m^2^/s) compared to OPP films (O_2_ permeability: 2.68 × 10^−12^ m^2^/s). Thus, the O_2_ decrease in PLA films was mostly due to its consumption by the fruit respiration.

### 3.3. Weight Loss, Firmness and Surface Color

**Figure 3:**
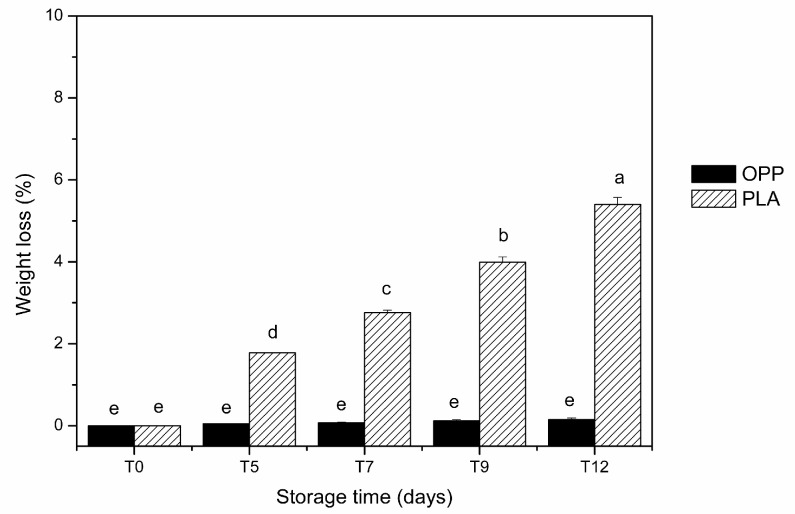
Changes in weight loss of packaged apple slices throughout 12 days of storage at 4 °C (OPP—oriented polypropylene; PLA—polylactic acid). Vertical bars indicate 95% confidence intervals of three replicates. Means with equal letters are not statistically different, as determined by Tukey’s test with a 5% significance level. T0—immediately after packaging; T2–T12—2–12 days after packaging and storage at 4 °C.

Due to their high water content (80–85%), Royal Gala apples present a high tendency to decrease in mass due to water loss caused by transpiration and respiration processes during storage. The weight loss of the whole package containing the fresh-cut apples in both package films was evaluated over 12 days of storage at 4 °C (Figure 3).

These results indicated that the percentage (%) of weight loss of the packaged apples in PLA film significantly increased (*p* < 0.05) with storage time, whereas no significant differences (*p* > 0.05) were observed in apples packaged in OPP film. After 12 days of refrigerated storage, the weight loss was higher in PLA films (5.4%) than in OPP films (0.2%) (Figure 3). These results demonstrated the higher permeability of PLA films to water, which was corroborated by the higher water vapor permeability of these biodegradable films (1.10 × 10^−12^ mol·m/m^2^·s·Pa) compared to OPP films (8.75 × 10^−14^ mol·m/m^2^·s·Pa). These results are in agreement with those reported by González-Buesa et al. [45], who described a weight loss of approximately 4% in fresh-cut celery after 21 days of storage using PLA compared to non-biodegradable bags. Zhou et al. [46] also observed a higher weight loss of fresh-cut melon when using PLA containers compared to PET.

**Figure 4:**
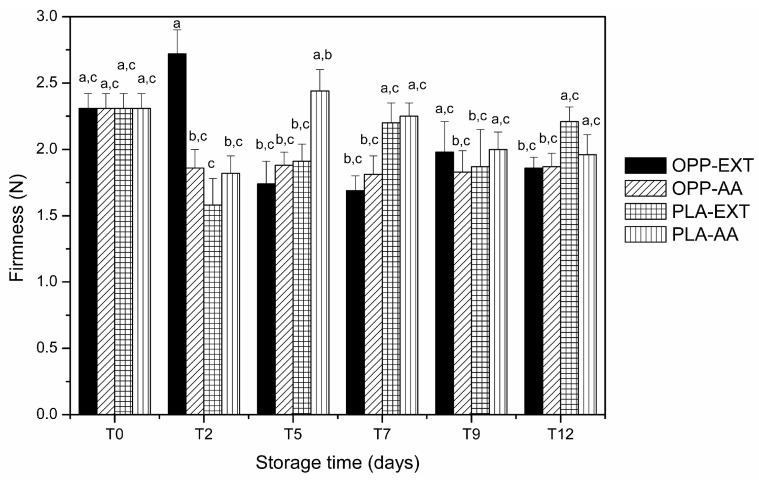
Changes in the firmness of packaged apple slices during 12 days of storage at 4 °C (OPP—oriented polypropylene; PLA—polylactic acid; EXT—extract of olive pomace; AA—ascorbic acid). Vertical bars indicate 95% confidence intervals of nine replicates. Means with equal letters are not statistically different, as determined by Tukey’s test with a 5% significance level. T0—immediately after packaging; T2–T12—2–12 days after packaging and storage at 4 °C.

Maintaining firmness is one of the most important physical attributes for ensuring the quality of fruits. In general, the firmness of fresh-cut apples appeared to be preserved, and no significant differences (*p* > 0.05) were observed between the firmness of apple slices stored in either package film and the tested antioxidant during storage at 4 °C (Figure 4). González-Buesa et al. [45] also reported that both PLA and petroleum-based plastic films maintained the firmness of celery during 14 days of storage.

The color of the fruit is another important organoleptic factor, as it can influence customer acceptance. The color characteristics of the fresh-cut apples incorporated with olive pomace extract and ascorbic acid and packaged with PLA and OPP films were evaluated as a function of storage time using the CIEL*a*b* color space (Table 1).

For all the samples prepared with olive pomace extract, L* was significantly reduced after two days (T2) of refrigeration, remaining stable throughout the storage time. However, for samples in OPP film (OPP-EXT), a decrease in L* was observed in the first five days (T5) of storage, and then remained constant. For samples prepared with ascorbic acid, no significant variation (*p* > 0.05) was observed in the fruits packaged in PLA film (PLA-AA) throughout 12 days of storage. In contrast, for the samples packaged in OPP film (OPP-AA), a slight decrease was observed only after five days (T5). After five days of refrigerated storage, significant differences (*p* < 0.05) in L* were detected between OPP and PLA in each antioxidant-treated apple. Based on the calculations, the samples at T0 presented the lowest values of Chroma (C), and refrigerated storage for two days (T2) promoted an increase of the samples’ brightness (Table 1). After this, the C-values of the tested samples seemed to be preserved, although a significant increase (*p* < 0.05) was observed at T9 (32.2 ± 0.5) and T12 (32.1 ± 0.4) for olive-pomace samples in OPP film (OPP-EXT). When comparing the different treatments with antioxidant solutions and the packaging during storage, the highest values were always observed for OPP-EXT. Regarding the hue angle (h°), T0 samples had higher values, ~100°, which characterized the yellow color of the apple slices. At T2, a slight but significant decrease (*p* < 0.05) was observed in the h° of all samples, which was maintained during the storage (Table 1), which could indicate a change to slight orange colors. The lowest values were perceived for samples with olive pomace using both films (OPP-EXT and PLA-EXT). Finally, the browning of the fruits was determined by the ΔE values obtained for each studied condition (Table 1). The results showed that after two days (T2) of refrigerated storage, an increase in the browning of the apple slices was noticed, which could be related to an increase in a* and b* values and the decrease in L*. After this, no variation in the browning of the apple slices was observed during storage (Table 1). Furthermore, no significant differences (*p* > 0.05) were observed between the packaging films when ascorbic acid was used. On the other hand, based on the significant differences (*p* < 0.05) between the browning for both films after two days of storage (7.07 ± 0.47 for PLA and 11.4 ± 0.7 for OPP) and throughout the storage time, the biodegradable PLA films seemed to be a better choice when using olive pomace extract as additive, showing that the natural extract may be more effective in packages with less O_2_. Similar results were obtained by Rocha and Morais [43], who also determined that two different periods of lightness decrease over time in Jonagored apples. The first period was observed in the first three days of storage, whin L* and h° decreased and a* increased due to the consumption of substrates by polyphenol oxidase, and the second period was described between the third and seventh day of storage as a preservation of browning. Furthermore, Song et al. [47] also described a marked change of color in fresh-cut apples during the early stage of storage that was maintained thereafter. During the minimal processing operations, such as cutting, an increase in respiration in the fruits is promoted. This process can induce the activation of polyphenol oxidase enzyme, which will react with the phenolic compounds to promote a loss of the natural color [48]. Furthermore, it is important to highlight that the initial browning of the samples using natural olive pomace extract (in the first two days of cold storage) can be associated with the natural color of the extract solution.

### 3.4. Microbial Load

Evaluating the microbial load is also crucial for assessing the quality and safety of fresh-cut fruits. The mesophilic bacteria, filamentous fungi and coliforms populations of the packaged fresh-cut apples packaged in both films, and after the addition of the tested antioxidants, were assessed immediately after different periods, specifically after packaging (T0) and after five days (T5) and 12 days (T12) of refrigerated storage (Table 2). This evaluation aimed to evaluate the effectiveness of PLA and OPP films and the performance of the olive pomace extract in promoting fruit quality compared to the commercial antioxidant ascorbic acid and the control samples.

The mesophilic bacteria population evaluated on the day of packaging and antioxidants addition (T0) was not significantly different (*p* > 0.05) between films, antioxidants and control samples, except for the control samples packaged in PLA film (4.63 ± 0.03 log CFU/g) (Table 2). The fresh-cut apples presented an aerobic mesophilic population ranging from 2.9 ± 0.2 log CFU/g to 4.63 ± 0.03 log CFU/g, which was in agreement with the previously reported results ranging from 2 to 4 log CFU/g [49,50]. On the other hand, the obtained counts were lower than those described by Graça et al. [51] for fresh-cut apple samples (3.3 to 8.9 log CFU/g). After storage for five and 12 days, the variation in mesophilic bacteria population in the packaged fresh-cut apples depended on the film packaging used. In general, the PLA film was more efficient in inhibiting bacteria growth (T5 and T12), for both olive pomace extract and ascorbic acid compared to the control samples. On the other hand, the use of olive pomace extracts seemed to inhibit bacteria growth during five days of storage (T5) compared to ascorbic acid, not only for PLA film (2.4 ± 0.1 log CFU/g using olive pomace extracts and 3.90 ± 0.04 log CFU/g using ascorbic acid), but also for OPP film (3.4 ± 0.1 log CFU/g using olive pomace extracts and 4.3 ± 0.1 log CFU/g using ascorbic acid). After 12 days of refrigerated storage, a significant (*p* < 0.05) increase in the bacterial contamination of apple slices was detected (Table 2). Concerning the legislation in Portugal, the recommended limits of mesophilic bacteria in ready-to-eat foods is 6 log CFU/g. Thus, neither of the control samples complied with these limits (5.7 ± 0.1 log CFU/g for OPP film and 5.8 ± 0.0 log CFU/g for PLA film). The samples using ascorbic acid as additive and OPP film as packaging bag also had higher bacterial contamination (5.6 ± 0.1 log CFU/g). Furthermore, it was possible to conclude that the olive pomace extract was able to delay the growth of bacteria after 12 days of storage using PLA film (3.75 ± 0.03 log CFU/g).

Regarding the filamentous fungi, the counts in fresh-cut apples were between 2.4 ± 0.1 and 4.44 ± 0.05 log CFU/g (Table 2). An increase in filamentous fungi counts was observed in the stored samples, except for the sample treated with olive pomace extract and packaged in PLA film (PLA-EXT) after five days of storage (2.44 ± 0.05 log CFU/g), which did not change from the beginning of the process (2.4 ± 0.1 log CFU/g). It was evident that olive pomace extracts were more efficient in inhibiting the growth of filamentous fungi compared to the samples treated with ascorbic acid and to control samples, particularly those sealed in PLA film. The counts of filamentous fungi population in ready-to-eat foods were limited to 2.7 log CFU/g, showing that the samples stored for five days in the PLA bag using the natural extract as additive could be considered of adequate quality in this regard.

Concerning coliforms, although the concentration complied with the recommended limits (4 log CFU/g), the results indicated that olive pomace extracts were able to inhibit the growth of coliforms, unlike ascorbic acid (Table 2). In a previous study, Graça et al. [51] had observed higher levels (between 1.8 and 7.6 log CFU/g) of coliforms in fresh-cut apples.

The overall results demonstrated that the use of olive pomace extracts could be more advantageous in inhibiting microbial growth in apple slices stored in either film than the samples treated with ascorbic acid. Furthermore, evaluating the different materials, PLA also showed higher inhibition of the microbial growth than OPP, and the storage for five days in cold temperatures could be proposed to ensure the quality of the fruits. The high antimicrobial activity of these extracts had been reported before by the authors, not only against Gram-negative and Gram-positive bacteria, but also against fungi [11,12]. Botondi et al. 2015 [52] and González-Buesa [45] described the same microbial growth trends when using both PLA and conventional plastics for fresh-cut produce packaging, which suggested that the results obtained in this work could be attributed to the combined effect of olive pomace extract and PLA film. In fact, the potential of PLA films as a suitable alternative to non-biodegradable plastics in preserving the quality and safety of fresh-cut fruits and vegetables was previously well-demonstrated [45,46,52,53].

### 3.5. Total Phenolic Content and Antioxidant Activity

The results of total phenolic content (TP) of the extracts of fresh-cut apples using water (control), olive pomace and ascorbic acid as antioxidants and packaged in PLA and OPP films, immediately after packaging (T0) and after two (T2), five (T5), seven (T7), nine (T9) and 12 days (T12) of storage, are presented in Table 3.

Phenolic compounds are important substances with significant contributions to the nutritional and sensory quality of fruits, and they present benefits to human and animal health. The higher values of TP for all the studied samples were observed at T12; although, when compared to T0, only a slight but significant (*p* < 0.05) increase in TP for samples with ascorbic acid (OPP-AA and PLA-AA) were detected. Nevertheless, no significant differences (*p* > 0.05) were noticed between the two antioxidants (olive pomace and ascorbic acid) and films (PLA and OPP) at T12. The overall results suggest that natural olive pomace extracts can be used as an ingredient to extend the shelf life of fresh-cut apples, particularly in terms of preserving TP during storage at refrigerated temperatures.

Table 4 shows the results concerning the antioxidant activity (measured by FRAP and DPPH assays) of the extracts of apple slices packaged in PLA and OPP films using water (control), olive pomace and ascorbic acid as antioxidants. The measurements were taken immediately after packaging (T0) and after two (T2), five (T5), seven (T7), nine (T9) and 12 days (T12) of storage, at a refrigerated temperature.

For the FRAP assay, a slight but significant (*p* < 0.05) increase was observed in the antioxidant activity of the samples at T12, except for the OPP-EXT sample (0.092 ± 0.003 mmol FSE/g extract). Nevertheless, both antioxidants demonstrated the ability to preserve or improve the antioxidant activity of the apple slices during storage. In the DPPH assay, the results were expressed as IC_50_ values, with higher values corresponding to lower antioxidant potentials (IC_50_: extract concentration corresponding to 50% of antioxidant activity). The antioxidant activity of the samples was preserved after 12 days of storage, with the exception of the samples treated with ascorbic acid and packaged in OPP film (OPP-AA), where a significant (*p* < 0.05) increase was noticed (IC_50_ value of 5.3 ± 0.2 mg/mL). Although the highest scavenging activity was obtained at T2 for all the samples, the overall results of antioxidant activity suggest that a storage of five days would be optimal to ensure the nutritional quality of the fruits, as supported by the obtained results of the microbial quality assays.

The increase in phenolic compounds and antioxidant activity may improve the functional value of the fresh-cut fruits, which can be explained by the cutting of the fruits before immersion in the antioxidant solutions and packaging. This process induces the synthesis and accumulation of phenolic compounds in a short time as a defense response. The cell damage in the wound area can promote physiological and biochemical changes, thus increasing the metabolic reactions and inducing phenylalanine ammonia-lyase (PAL) activity, which enhances phenolic accumulation and, consequently, antioxidant activity in the apples [54].

### 3.6. Principal Component Analysis and Clustering

After the one variable at a time (OVAT) approach, two multivariate analysis techniques (PCA and K-means) were applied to understand the overall contribution of each part of the data collected and attached in the repository linked to this publication. The final dataset was clean and considered the means of measurement replicates while maintaining experimental replicates. Afterwards, an imputation of data was performed when necessary, and only complete cases of observations/variables were considered. Finally, the data were scaled to avoid numeric instabilities. Since the microbiological quality of the packaged fresh-cut apples was only studied for storage periods of zero, five and 12 days, and for easier interpretation of the results, the PCA analysis was performed only for these periods. In addition, some variables with lower statistical weight were removed from the analysis.

**Figure 5:**
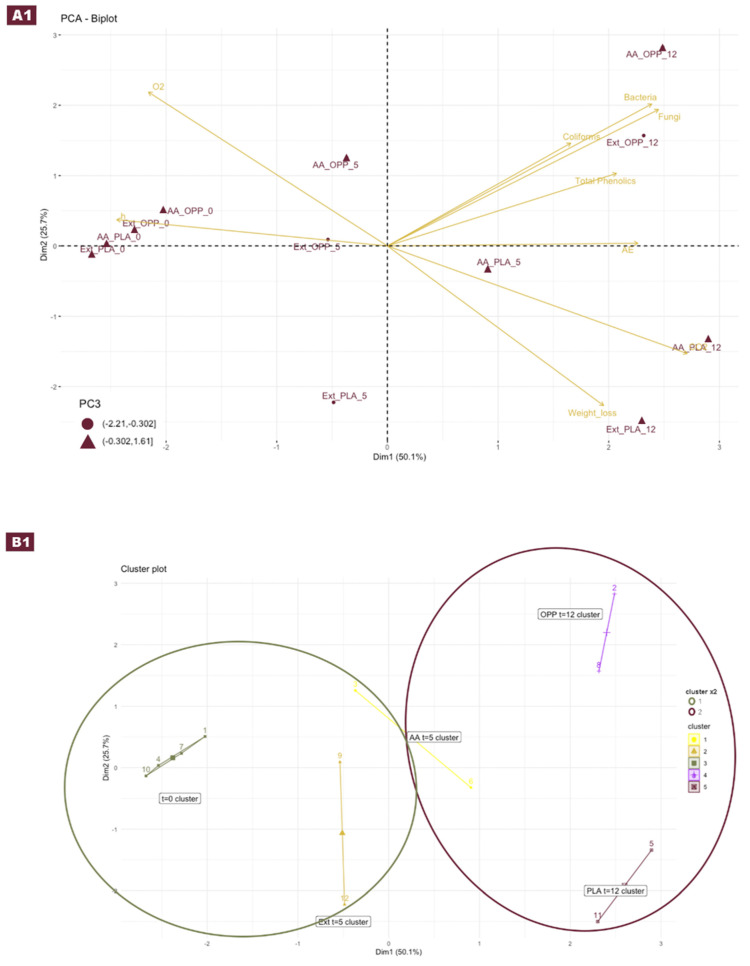
Principal component analysis with (**A1**) biplot visualization of the three main principal components, and K-means superimposed layer with (**B1**) cluster plot representing the K2 and K5 ranges (OPP—oriented polypropylene; PLA—polylactic acid; EXT—extract of olive pomace; AA—ascorbic acid).

In Figure 5, a visual representation of both analyses is displayed and divided into two sections: section A shows the PCA analysis results, and section B exhibits the K-means clustering results (for numerical representation of data, please see the Supplementary Materials). The scree plot for the PCA analysis shows that the two first principal components accounted for approximately 75% of the total variance, and inclusion of the third component would increase the explained variance to approximately 90%. Therefore, by employing the loadings of the three main principal components (PC), a biplot with a two-dimensional plot representation of the PC1 and PC2 can be plotted (Figure 5(A1)). A third PC with a different marker shape was also represented, considering the negative range of numbers with a circular shape and the positive range of values with a triangular shape.

To select the optimal number of clusters for the K-means technique, the analysis relied on the silhouette method, which suggests the higher point in the average silhouette width (five in this work). Nevertheless, to further explore the possible cluster combinations, two clusters were also considered. Therefore, in the subsection plot B1 (Figure 5(B1)), the final PCA overlay data is presented in both two (circles) and five (straight lines polygons) clusters. The two K-means clusterizations suggested a separation between samples collected at day zero and day five (except for one observation of ascorbic acid treatment), emphasizing more notorious changes over longer storage times (12 days). On the other hand, when clustering with five centers, a clear separation of the treatments (EXT vs. AA) was observed for day five and, there was also a separation at day 12 based on the type of bag in which the samples were stored.

Considering plot A1 (Figure 5(A1)) as the core of the combined analysis, the variables that influenced each of the principal components could be identified. Hence, PC1 or the X-axis attributes the higher variance to the CO_2_, bacteria and fungi to the right and O_2_ and hue angle (h°) parameters to the left. Also, it is important to note that apple samples of the same antioxidant and polymer-bag treatments started to the left, and the time increments moved the observations to the right. On the PC2 or Y-axis, the higher positive variables spotted were bacteria, fungi and O_2_, while the higher negative variables were weight loss and CO_2_, demonstrating a negative correlation (please see Apples_EDA plot included in the repository) to O_2_ (−0.86). It is worth noting that the reported weight loss refers to the weight loss of the samples, antioxidant, polymer bag and gases complex, and not only to apple weight loss. On the other hand, the coliforms variable represented a significant separator in the PC2 due to the absence of growth in the extract-treated samples compared to an average of 2 log CFU/g from the ascorbic acid-treated samples for samples with storage times higher than T0. Additionally, in the PC3, it was possible to better visualize the obtained clusters in plot B1, which showed a clear difference between the different bag materials after 12 days of storage.

## 4. Conclusions

The demand for fresh-cut fruits with preservation of their quality attributes has been increasing in recent years. Therefore, the food industry is always seeking out new functional products that satisfy the needs and tastes of consumers who are concerned about the choice of healthier foods. This work revealed that natural olive pomace extracts could be used as an ingredient in fresh-cut apples to increase the antioxidant activity of the fruits and to inhibit microbial growth during storage at refrigerated temperatures for at least five days. In addition, these natural extracts were demonstrated to preserve the color of the apple slices after the initial browning of the samples. This can be attributed to the natural color of the olive pomace extract solution. Concerning the packaging films used, PLA films seemed to exhibit a higher potential to preserve the quality of fresh-cut apples in combination with olive pomace extracts used as an additive compared to conventional films. Although it is also dependent on many factors related to the harvesting and postharvest handling of fresh produce, the promising results obtained in this work supported the antimicrobial potential of olive pomace extracts, suggesting that their use as an antioxidant could be more suitable for fresh-cut products in which the color is not a conditional factor for the consumer’s evaluation and consumption decisions. The recovery of functional compounds from olive waste is in line with consumers’ requirements for high-quality and safe processed foods. At the same time, it can reduce environmental impacts, also promoting the sustainability of the olive oil industries. Further investigation should be performed in order to understand the possible interactions between the organoleptic profile of the samples, assessed by a sensory analysis to evaluate the quality and the potential commercial acceptance of these new products. Furthermore, the possibility of incorporating these extracts into food packaging should continue to be explored to reduce food waste.

## Figures and Tables

**Table 1:** Color parameters of PLA and OPP packaged fresh-cut apples for 12 days at 4 °C.

		L*					
		T0	T2	T5	T7	T9	T12
OPP	EXT	79.7 ± 0.2 ^a,A^	77.09 ± 0.47 ^b,B^	74.3 ± 0.3 ^c,C^	74.9 ± 0.4 ^c,C^	74.04 ± 0.47 ^c,C^	74.3 ± 0.5 ^c,C^
	AA	79.4 ± 0.2 ^a,A^	78.6 ± 0.3 ^a,b,A^	77.4 ± 0.3 ^b,c,B^	76.9 ± 0.4 ^c,B^	77.2 ± 0.4 ^c,B^	76.8 ± 0.3 ^c,B^
PLA	EXT	79.7 ± 0.2 ^a,A^	76.97 ± 0.22 ^b,c,B^	76.1 ± 0.5 ^c,B^	77.3 ± 0.3 ^b,c,A,B^	77.6 ± 0.3 ^b,A,B^	76.7 ± 0.3 ^b,c,B^
	AA	79.4 ± 0.2 ^a,A^	78.5 ± 0.3 ^a,A^	78.9 ± 0.2 ^a,A^	78.5 ± 0.2 ^a,A^	78.6 ± 0.3 ^a,A^	78.7 ± 0.3 ^a,A^
		**C**					
		T0	T2	T5	T7	T9	T12
OPP	EXT	20.9 ± 0.2 ^c,A^	29.1 ± 0.6 ^b,A^	28.6 ± 0.4 ^b,A^	29.4 ± 0.5 ^b,A^	32.2 ± 0.5 ^a,A^	32.1 ± 0.4 ^a,A^
	AA	19.7 ± 0.2 ^c,B^	24.9 ± 0.5 ^a,b,B^	22.9 ± 0.5 ^b,C^	24.1 ± 0.5 ^a,b,C^	23.5 ± 0.5 ^b,C^	25.3 ± 0.4 ^a,C^
PLA	EXT	20.9 ± 0.2 ^b,A^	26.6 ± 0.5 ^a,B^	25.8 ± 0.5 ^a,B^	25.8 ± 0.4 ^a,B^	26.1 ± 0.3 ^a,B^	27.01 ± 0.40 ^a,B^
	AA	19.7 ± 0.2 ^d,B^	25.4 ± 0.5 ^a,B^	23.2 ± 0.4 ^b,c,C^	22.8 ± 0.3 ^c,C^	23.1 ± 0.4 ^b,c,C^	24.5 ± 0.4 ^a,b,C^
		**h°**					
		T0	T2	T5	T7	T9	T12
OPP	EXT	99.3 ± 0.1 ^a,A^	87.6 ± 0.4 ^c,D^	89.3 ± 0.3 ^b,C^	89.4 ± 0.4 ^b,C^	89.3 ± 0.3 ^b,C^	87.9 ± 0.3 ^b,c,B^
	AA	99.6 ± 0.3 ^a,A^	93.4 ± 0.4 ^b,B^	93.5 ± 0.4 ^b,A^	92.7 ± 0.4 ^b,A^	92.6 ± 0.4 ^b,A^	92.7 ± 0.3 ^b,A^
PLA	EXT	99.3 ± 0.1 ^a,A^	91.6 ± 0.3 ^b,C^	91.5 ± 0.3 ^b,B^	90.7 ± 0.2 ^b,B^	90.7 ± 0.3 ^b,B^	89.5 ± 0.4 ^c,B^
	AA	99.6 ± 0.3 ^a,A^	95.8 ± 0.4 ^b,A^	94.7 ± 0.3 ^b,c,A^	93.6 ± 0.2 ^c,d,A^	93.5 ± 0.3 ^c,d,A^	93.1 ± 0.4 ^d,A^
		**ΔE**					
		T0	T2	T5	T7	T9	T12
OPP	EXT	-	11.4 ± 0.7 ^a,b,A^	10.3 ± 0.5 ^b,A^	11.8 ± 0.4 ^a,b,A^	11.8 ± 0.7 ^a,b,A^	14.1 ± 0.7 ^a,A^
	AA	-	5.1 ± 0.6 ^a,B^	4.3 ± 0.5 ^a,C^	5.4 ± 0.6 ^a,C,B^	5.2 ± 0.5 ^a,B,C^	6.4 ± 0.5 ^a,B,C^
PLA	EXT	-	7.07 ± 0.47 ^a,B^	7.4 ± 0.7 ^a,B^	6.3 ± 0.5 ^a,B^	6.7 ± 0.3 ^a,B^	8.02 ± 0.46 ^a,B^
	AA	-	5 ± 1 ^a,B^	4.2 ± 0.5 ^a,C^	4.01 ± 0.30 ^a,C^	4.7 ± 0.6 ^a,C^	5.5 ± 0.5 ^a,C^

PLA—polylactic acid; OPP—oriented polypropylene; EXT—extract of olive pomace; AA—ascorbic acid; T0—immediately after packaging; T2–T12—2–12 days after packaging and storage at 4 °C. Results are presented as means ± standard error. For each color parameter, the means in each row with the same letters (^a–d^) and the means in each column with the same letters (^A–D^) are not significantly different (*p* > 0.05).

**Table 2:** Microbial load on PLA- and OPP-packaged fresh-cut apples after 12 days of storage at 4 °C.

		Mesophilic Bacteria (Log CFU/g)			Filamentous Fungi (Log CFU/g)			Coliforms (Log CFU/g)		
		T0	T5	T12	T0	T5	T12	T0	T5	T12
OPP	EXT	3.5 ± 0.1 ^b,B^	3.4 ± 0.1 ^b,D^	5.2 ± 0.1 ^a,A^	2.5 ± 0.1 ^c,C^	3.3 ± 0.1 ^b,C^	5.25 ± 0.05 ^a,A^	n.d.	n.d.	n.d.
	AA	3.2 ± 0.1 ^c,B,C^	4.3 ± 0.1 ^b,B^	5.6 ± 0.1 ^a,A^	3.2 ± 0.1 ^c,B^	4.03 ± 0.09 ^b,B^	5.25 ± 0.04 ^a,A^	n.d.	2.0 ± 0.3 ^b,A^	3.1 ± 0.1 ^a,A^
	NT	3.3 ± 0.1 ^c,B,C^	5.2 ± 0.1 ^b,A^	5.7 ± 0.1 ^a,A^	2.9 ± 0.2 ^c,B,C^	>6 ^a,A^	5.18 ± 0.04 ^b,A^	2.2 ± 0.1 ^a^	2.2 ± 0.1 ^a,A^	n.d.
PLA	EXT	2.9 ± 0.1 ^b,B,C^	2.4 ± 0.1 ^c,E^	3.75 ± 0.03 ^a,C^	2.4 ± 0.1 ^b,C^	2.44 ± 0.05 ^b,D^	3.3 ± 0.1 ^a,C^	n.d.	n.d.	n.d.
	AA	2.9 ± 0.1 ^c,C^	3.90 ± 0.04 ^b,C^	4.2 ± 0.1 ^a,B^	2.8 ± 0.3 ^b,B,C^	3.84 ± 0.03 ^a,B^	4.2 ± 0.2 ^a,B^	n.d.	1.8 ± 0.1 ^a,A^	1.6 ± 0.2 ^a,B^
	NT	4.63 ± 0.03 ^b,A^	4.5 ± 0.1 ^b,B^	5.80 ± 0.01 ^a,A^	4.44 ± 0.05 ^b,A^	4.02 ± 0.07 ^c,B^	5.3 ± 0.1 ^a,A^	n.d.	1.5 ± 0.1 ^a,A^	1.3 ± 0.3 ^a,B^

PLA—polylactic acid; OPP—oriented polypropylene; EXT—extract of olive pomace; AA—ascorbic acid; NT—non-treated samples; T0—immediately after packaging; T5—5 days after packaging and storage at 4 °C; T12—12 days after packaging and storage at 4 °C; n.d.—not detected. Results are presented as means ± standard error. For each microbial group, the means in each row with the same letters (^a–c^) and means in each column with the same letters (^A–E^) are not significantly different (*p* > 0.05).

**Table 3:** Total Phenolic Content of PLA and OPP packaged fresh-cut apples for 12 days at 4 °C.

		Mg GAE/g Extract					
		T0	T2	T5	T7	T9	T12
OPP	EXT	5.4 ± 0.2 ^a,b,c,A^	4.9 ± 0.2 ^b,c,A^	4.6 ± 0.1 ^c,A,B^	4.7 ± 0.1 ^b,c,A^	5.4 ± 0.2 ^a,b,A^	6.1 ± 0.3 ^a,A,B^
	AA	5.7 ± 0.4 ^b,A^	4.8 ± 0.2 ^b,c,A^	4.4 ± 0.2 ^c,B^	4.5 ± 0.2 ^c,A^	5.1 ± 0.3 ^b,c,A^	7.0 ± 0.2 ^a,A^
	NT	4.68 ± 0.07 ^a,b,A^	3.5 ± 0.1 ^c,B^	4.08 ± 0.15 ^b,c,B^	4.42 ± 0.01 ^a,b,c,A,B^	5.01 ± 0.22 ^a,b,A^	5.4 ± 0.5 ^a,B^
PLA	EXT	4.9 ± 0.4 ^a,b,A^	5.0 ± 0.2 ^a,b,A^	4.3 ± 0.1 ^b,c,B^	3.8 ± 0.1 ^c,B^	4.9 ± 0.2 ^a,b,A^	5.8 ± 0.3 ^a,B^
	AA	5.0 ± 0.2 ^b,A^	4.6 ± 0.2 ^b,A,B^	5.3 ± 0.2 ^b,A^	5.0 ± 0.2 ^b,A^	5.04 ± 0.15 ^b,A^	6.5 ± 0.2 ^a,A,B^
	NT	4.5 ± 0.3 ^b,c,A^	3.70 ± 0.01 ^d,A,B^	4.48 ± 0.07 ^b,c,A,B^	5.01 ± 0.00 ^a,b,A^	4.14 ± 0.06 ^c,d,A^	5.53 ± 0.09 ^a,B^

PLA—polylactic acid; OPP—oriented polypropylene; EXT—extract of olive pomace; AA—ascorbic acid; NT—non-treated samples; GAE—gallic acid equivalents; T0—immediately after packaging; T2–T12—2–12 days after packaging and storage at 4 °C. Results are presented as means ± standard error. The means in each row with the same letters (^a–d^) and means in each column with the same letters (^A–B^) are not significantly different (*p* > 0.05).

**Table 4:** Antioxidant activity measured by FRAP and DPPH assays of PLA and OPP packaged fresh-cut apples for 12 days at 4 °C.

		mmol FSE/g Extract					
		T0	T2	T5	T7	T9	T12
OPP	EXT	0.078 ± 0.004 ^b,A^	0.089 ± 0.006 ^a,b,A,,B^	0.098 ± 0.002 ^a,B^	0.091 ± 0.003 ^a,b,B^	0.079 ± 0.006 ^b,A^	0.092 ± 0.003 ^a,b,B,C^
	AA	0.080 ± 0.005 ^c,A^	0.087 ± 0.003 ^b,c,A,B^	0.098 ± 0.003 ^a,b,B^	0.089 ± 0.003 ^b,c,B^	0.091 ± 0.005 ^a,b,c,A^	0.104 ± 0.001 ^a,A^
	NT	0.0756 ± 0.0001 ^c,A^	0.075 ± 0.003 ^c,A^	0.096 ± 0.003 ^a,B^	0.085 ± 0.002 ^b,B,C^	0.082 ± 0.002 ^b,c,A^	0.0862 ± 0.0004 ^b,C^
PLA	EXT	0.076 ± 0.002 ^b,c,A^	0.096 ± 0.005 ^a,A^	0.093 ± 0.003 ^a,b,B^	0.075 ± 0.003 ^c,C^	0.082 ± 0.005 ^a,c,A^	0.096 ± 0.005 ^a,A,B,C^
	AA	0.079 ± 0.002 ^d,A^	0.088 ± 0.003 ^c,d,A,B^	0.117 ± 0.003 ^a,A^	0.103 ± 0.001 ^b,A^	0.089 ± 0.002 ^c,A^	0.100 ± 0.001 ^b,A,B^
	NT	0.07623 ± 0.00005 ^c,A^	0.065 ± 0.003 ^d,B^	0.100 ± 0.001 ^a,B^	0.094 ± 0.003 ^a,b,A,B^	0.0683 ± 0.0004 ^c,d,A^	0.0890 ± 0.0001 ^b,B,C^
		IC_50_ (mg/mL)					
		T0	T2	T5	T7	T9	T12
OPP	EXT	6.3 ± 0.2 ^a,B^	4.3 ± 0.1 ^c,D^	4.0 ± 0.1 ^c,D^	5.3 ± 0.1 ^b,C^	4.3 ± 0.1 ^c,D^	6.4 ± 0.1 ^a,B,C^
	AA	7.8 ± 0.2 ^a,A^	4.5 ± 0.1 ^c,C,D^	5.7 ± 0.1 ^b,B^	5.3 ± 0.2 ^b,C^	7.2 ± 0.2 ^a,A^	5.3 ± 0.2 ^b,D^
	NT	7.12 ± 0.04 ^a,b,A^	6.2 ± 0.1 ^c,d,B^	5.7 ± 0.2 ^d,B^	6.6 ± 0.1 ^b,c,A,B^	4.16 ± 0.05 ^e,D^	7.5 ± 0.1 ^a,A^
PLA	EXT	7.4 ± 0.1 ^a,A^	4.7 ± 0.1 ^d,C^	6.8 ± 0.1 ^b,A^	7.4 ± 0.1 ^a,A^	5.7 ± 0.1 ^c,C^	7.0 ± 0.1 ^a,b,A,B^
	AA	5.5 ± 0.1 ^a,C^	4.4 ± 0.1 ^b,C,D^	4.1 ± 0.1 ^b,C,D^	5.1 ± 0.1 ^a,C^	4.4 ± 0.1 ^b,D^	5.4 ± 0.2 ^a,D^
	NT	7.3 ± 0.1 ^b,A^	8.2 ± 0.1 ^a,A^	4.6 ± 0.1 ^e,C^	6.3 ± 0.1 ^c,B^	6.3 ± 0.2 ^c,B^	5.7 ± 0.1 ^d,C,D^

PLA—polylactic acid; OPP—oriented polypropylene; EXT—extract of olive pomace; AA—ascorbic acid; NT—non-treated samples; FES—ferrous sulfate equivalents; T0—immediately after packaging; T2–T12—2–12 days after packaging and storage at 4 °C. Results are presented as means ± standard error. For each assay, means in each row with the same letters (^a–e^) and means in each column with the same letters (^A–D^) are not significantly different (*p* > 0.05).

## Data Availability

The data presented in this study are available on request from the corresponding author.

## Supplementary Materials

The following supporting information can be downloaded at: https://www.mdpi.com/article/10.3390/foods12091926/s1, Figure S1: PCA and K-means Data are available online at https://github.com/Bruno-melgar/Apples (accessed on 30 March 2023).
