# Supplementary figures and images for: Effect of Olive Pomace Extract Application and Packaging Material on the Preservation of Fresh-Cut Royal Gala Apples
Source: Foods. 2023 May 8;12(9):1926. doi: 10.3390/foods12091926 (PMC10178254; doi:10.3390/foods12091926)

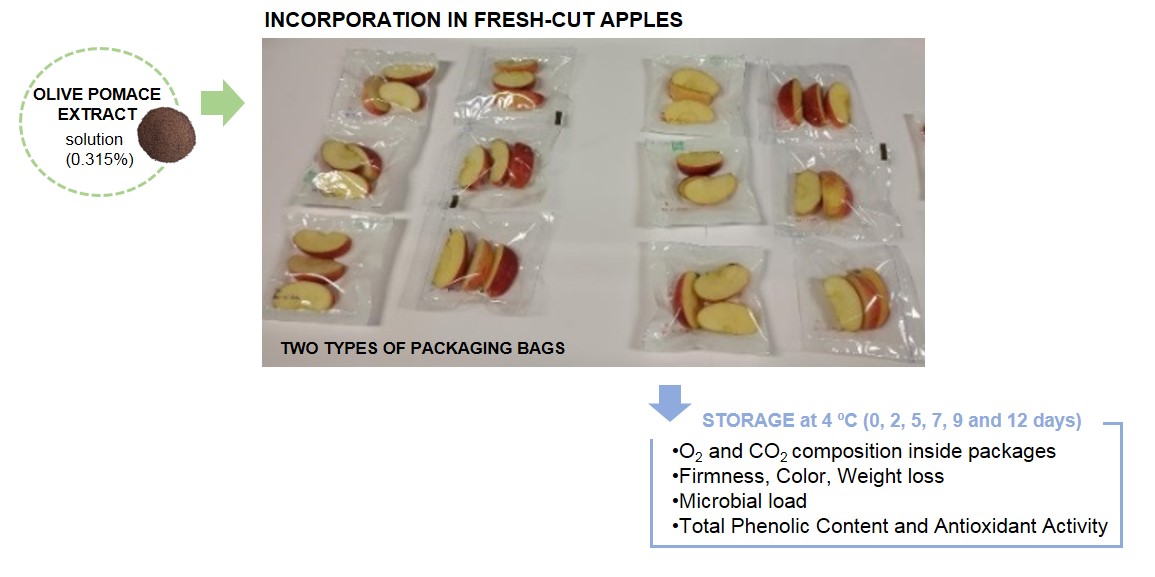

Supplement: Supplementary file 1 [file foods-12-01926-s001.zip › Supplementary Figure S1.jpg]
